# Radiation Oncologists’ Approach to Rectosigmoid Junction Tumors in Turkey: The Turkish Society for Radiation Oncology Gastrointestinal Group Survey Study (TROD 02-007)

**DOI:** 10.5152/tjg.2023.22597

**Published:** 2023-09-01

**Authors:** Özlem Aynacı, Lasif Serdar, Emine Canyılmaz, Pervin Hürmüz

**Affiliations:** 1 Department of Radiation Oncology, Karadeniz Technical University Faculty of Medicine, Trabzon, Turkey; 2 Department of Radiation Oncology, Kanuni Research and Education Hospital, Trabzon, Turkey; 3 Department of Radiation Oncology, Hacettepe University Faculty of Medicine, Ankara, Turkey

**Keywords:** Survey, rectosigmoid junction, surgery, neoadjuvant chemoradiotherapy

## Abstract

**Background/Aims:**

The objective was to determine the preferences and perspectives regarding preoperative evaluation and treatment strategies for rectosigmoid junction cancer among radiation oncologists using a questionnaire survey.

**Materials and Methods:**

Since defining the correct origin of the neoplasm is essential in tailoring the most appropriate treatment scheme in the rectosigmoid junction region, we surveyed Turkish radiation oncologists about clinical decisions in rectosigmoid junction cancer patients via a 20-point questionnaire.

**Results:**

Based on responses from 130 radiation oncologists surveyed across the country, 119 (91.5%) used the anterior peritoneal reflection as the landmark, while 111 (85.4%) used the distance from the anal verge to the boundary between the rectum and sigmoid. This indicates that most of the participants decided to consider both pretreatment evaluation methods. Although distance at colonoscopy can be very variable, when the discrepancy was observed between these methods, 66 (50.8%) participants made the final decision according to the distance from the anal verge in our questionnaire. The conclusion from the questionnaire is that there is difficulty in interpreting magnetic resonance imaging findings, and there is a need for anatomic landmarks relevant to the limit between the rectum and sigmoid so that clinicians can confidently facilitate the diagnosis.

**Conclusions:**

There is a wide variation in the diagnosis and decision-making of rectosigmoid junction cancer among radiation oncologists in Turkey, according to our survey, because of several definitions of the rectosigmoid junction boundaries. Considerable attention is required to clarify whether the first preoperative treatment or surgery for rectosigmoid junction cancer.

**Main Points**

- Radiation oncologists have significantly more fear of making decision on rectosigmoid junction (RSJ) cancer in regards to the difference in approach between colon and rectal cancer.
- Radiological examinations such as magnetic resonance imaging findings should be studied carefully, and the localization of the sigmoid colon above or below the line of the promontorium should be considered.
- During delineating target volumes for radiotherapy, physicians should get more training especially RSJ according to the peritoneal reflection.

## Introduction

Colorectal cancers are the third most frequently occurring after breast and lung cancers, and approximately 1/3 of colorectal cancers are rectal cancers.^1^ Patients with non-metastatic clinical stage T3/T4 disease and/or rectal cancer with regional lymph node involvement with a high risk of recurrence are defined as locally advanced rectal cancer (LARC). Thirty years ago, the treatment of rectal cancer was only surgery. The necessity of postoperative chemoradiotherapy (CRT) was revealed after local recurrence rates were between 45% and 65% in LARC.^2^ However, the increase in local recurrence rates in patients with positive surgical margins receiving postsurgical CRT suggested neoadjuvant treatment. Trials showed that preoperative CRT, compared with postoperative CRT, improved local control and was associated with reduced toxicity but did not improve overall survival.^3^

The last part of the large intestine, which ends with the anus, is called the rectum, and the left, that is, the last part of the descending colon, is called the sigmoid colon. And, the rectosigmoid junction (RSJ) is defined as the part of the large bowel between the sacral promontory and the lower margin of the second sacral vertebra where the sigmoid mesocolon terminates. Although it is anatomically considered part of the sigmoid colon, the RSJ is provided through a surgically important vascular system with the rectum above the peritoneal reflection, suggesting it would be better considered as part of the rectum.^4^ Cancer in the sigmoid and proximal rectum is important on behalf of requires different therapeutic approaches. Currently, there is no further available data regarding approaches to treating RSJ cancer. More accurate radiological staging, multimodal therapeutic interventions, improved surgical and radiation therapy (RT) techniques, and more comprehensive histopathological evaluation have all contributed to patient management and survival.^5^

This survey aims to report the tendencies in the pretreatment evaluation of RSJ and to identify the factors that influence the decision for treatment among radiation oncologists (RO) in Turkey. These data may lead to the need for new evidence-based criteria for RSJ cancer.

## Materials and Methods

From February to April, a questionnaire survey on RSJ cancer was carried out among 405 RO specialists via Google forms to research the day-to-day practice of RSJ treatment in Turkey. The survey reached 130 ROs, resulting in a 32% response rate. The questionnaire comprised 20 questions and was mainly divided into 3 parts to evaluate (i) demographic characteristics in participants, (ii) preoperative imaging methods, and (iii) treatment approaches. Participating specialists were asked questions relating to preoperative imaging methods, tumor localization assessment, and treatment strategy decision-making. The survey form was prepared by the researchers taking into account the studies in the literature consisting of 20 questions examining the knowledge and experience of specialist physicians in preoperative imaging procedures and treatment planning for rectum, rectosigmoid, and sigmoid colon cancers. This study approval is obtained from the ethical committee of Karadeniz Technical University, Faculty of Medicine, Farabi Hospital Ethics/Institutional Review Board (No.24237859-784).

### Statistical Analysis

Data were recorded and analyzed for statistical analyses on Statistical Package for the Social Sciences Statistics software version 23 (IBM corp., Armonk, NY, USA). While the frequency distributions were calculated for the categorical variables, the chi-square test was used to determine relationships between categorical variables. The statistical significance level was *P *< .05.

## Results

### Demographic Characteristics

One hundred thirty participated in the survey, with variable responses to questions. The survey items and responses are shown in Table 1. The majority of respondents were 40 years and older (63.3%). Fifty-nine (45.3%) worked in a university hospital compared with 45 (34.6%) in an education-research hospital, 19 (14.6%) in a private setting, and 7 (5.4%) in a community hospital. According to their clinical experience, 88 (67.7%) respondents treated ≤5 patients per month, and 42 (32.3%) participants >5 patients per month with a diagnosis of RSJ cancer. In addition, 53 (41.8%) respondents (associate professors and professors were 23.3% and 18.5%, respectively) worked in an academic environment.

### Preoperative Imaging Methods

**Figure 1.**
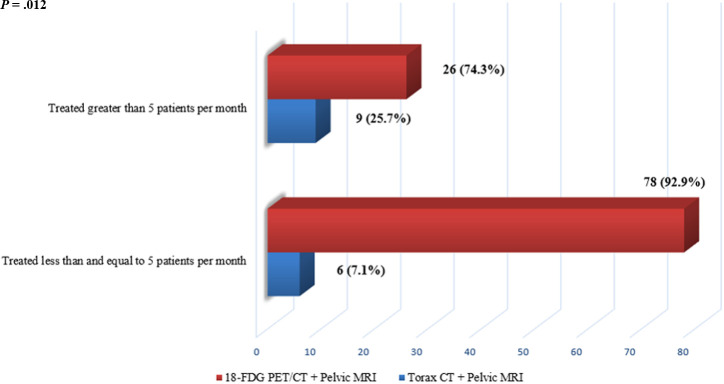
The question was related to using 18-FDG PET/CT and pelvic MRI to delineate the target volumes for radiotherapy of RSJ cancer patients. This graph represents responses compared to the number of participants treating more or less than 5 patients per month. 18-FDG PET/CT, 18 fluorodeoxyglucose-positron emission tomography/computed tomography; MRI, magnetic resonance imaging; RSJ, rectosigmoid junction.

For the 109 (83.8%) respondents, the decision to manage the treatment process was made in multidisciplinary tumor boards, considering the implementation of surgery and the necessity for neoadjuvant/adjuvant treatments. In 108 (83.1%) participants, responses were stated as using a combination of 18 fluorodeoxyglucose (18-FDG)-positron emission tomography-computed tomography (PET/CT) and contrast-enhanced magnetic resonance imaging (MRI) for the pre-RT staging of the disease. Diffusion-weighted MRI was added to standard MRI for 56 (43%) respondents considering better treatment response and tumor volume delineation. Colonoscopy reports were considered primarily by the 111 (85.4%) respondents for pretreatment evaluation rather than radiologic examinations. When compared to the number of patients treated per month, patients who treated less than 5 patients per month used 18-FDG PET/CT in addition to pelvic MRI to delineate the target volumes for radiotherapy of RSJ cancer patients (*P* = .012)(Figure 1).

When the survey was examined, the previous answer did not limit answering the following questions (11-14) regarding decision-making before the treatment. However, in question 16, it was emphasized which examination was taken into consideration primarily while making the final decision. The number of RO who evaluated the tumor’s location according to the peritoneal reflection in MRI was 119 (91.5%). Furthermore, 111 (85.4%) of the clinicians made the preoperative CRT decision according to the distance of the tumor from the anal verge. It seems that the participants take both into account. If participants were undecided in assessing, 66 (50.8%) RO chose treatment modality according to how far from the anal verge distance in colonoscopy without minding whether the tumor is above the peritoneal reflection in the lower abdomen CT and MRI. Regarding approaches with RSJ and upper rectum tumors, they recommended surgery first considering the similarity of the disease localization to colon cancer, with 31 (23.8%) and 21 (16.4%), respectively.

### Treatment Approaches

**Figure 2.**
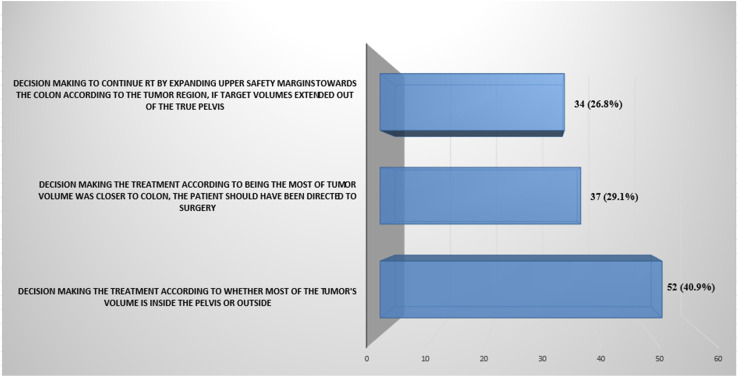
The question was related to different approaches that were taken according to the tumor region during the RT contouring. Each bar represents different responses from the participants. RT, radiation therapy.

In the present study, physicians’ responses showed a remarkable variety of treatment approaches for RSJ cancer. Preoperative CRT consequently surgery was selected in 39.2% of participants, and preoperative CRT consequently chemotherapy followed by surgery was selected in 32.3% of participants. Eleven (8.6%) participants emphasized that their treatment decision was based on whether most of the tumor volume was in the sigmoid or rectum. Moreover, in upper rectum cancer, it was observed that approximately half of RO answered this question (55%) tending surgery after neoadjuvant CRT. During the RT contouring, different approaches were observed by the participants. Fifty-two (40.9%) respondents commented that even if target volumes extended out of the true pelvis, it was continued by expanding upper safety margins toward the colon according to the tumor region; 37 (29.1%) respondents reported that being the most tumor volume was closer to the colon, the patient should have been directed to surgery. Thirty-four (26.8%) respondents planned the treatment according to whether most of the tumor’s volume was inside the pelvis or outside (Figure 2). Volumetric arc therapy was the most frequently used radiation technique (71%). Only (18.3%) of the respondents used 3-dimensional conformal radiotherapy (3DCRT) in the RSJ cancer treatment. Considering whether there was a difference in treatment approaches during coronavirus disease 2019 (COVID-19) pandemic, this study showed that the majority explained that there were no differences in the treatment decisions for 106 (82.2%) clinicians in this process, the decision taken as hypofractionated RT in 11 (6.9%) and 11 (10.9%) clinicians were primarily directed to surgery due to the tumor was already in a contradictory region such as the RSJ.

## Discussion

This survey has attempted to ascertain current Turkish ROs’ preferences and views in dealing with RSJ cancer in Turkey. For surgeons, visible morphological findings such as lateral bulges called haustra and fatty projections called appendices epiploicae yield more precise results, but RO cannot use reliable radiological landmarks. The problem is that no definition indicates the same anatomical region, which can be misleading.

Locally advanced rectal cancer requires precise preoperative tumor staging and requires a multimodality approach of systemic chemotherapy, RT, and total mesorectal excision to provide excellent survival and organ preservation rates.^6^ Treatment of colon cancer is based mainly on the cancer stage, and surgery is the primary treatment. Surgery to remove the portion of the colon containing cancer (partial colectomy) and nearby lymph nodes may be the only treatment needed, followed by chemotherapy.^7^ The colon is approximately 150 cm of the gastrointestinal tract between the ileocecal valve and the rectosigmoid corner. The sigmoid colon is the continuation of the descending colon, and while its length is variable, it is approximately 40 cm.^8^ The RSJ, the limit between the sigmoid colon and the upper rectum, is essential in surgery for functional and RT for tumoral diseases.^9^ Falch et al^10^ investigated more information about tumor characteristics and post-therapeutic consequences among the carcinomas of the RSJ, the sigmoid colon, or the upper rectum. As a result, pathologic findings such as tumor ulceration and polypoid tumor growth resembled rectal cancer. The RSJ region was a worse survival factor, whereas the other locations were not (*P* = .01).

Multidisciplinary tumor board meetings are joint in Turkey. This may show that only the majority of participants benefit from specific multidisciplinary discussions. A previous study about tumor boards’ impact on rectal cancer has established a statistically significant survival benefit in patients with colorectal cancer who had undergone multidisciplinary tumor board evaluation compared with those who had not.^11^ The responders stated that treatment approaches (83.8%) were mostly decided on multidisciplinary tumor boards about RSJ cancer.

The accuracy of MRI for staging rectal cancer has been reported to be between 31% and 100% for the T stage and 39% and 95% for the nodal stage.^12^ The National Comprehensive Cancer Network guidelines recommend 18-FDG PET/CT in patients with potentially surgically curable M1 disease and considered for image-guided liver-directed therapies for liver metastases; however, the routine use of 18-FDG PET/CT in colorectal cancer has not been recommended until now. The 18-FDG PET/CT has been shown to be applicable in accurately delineating tumors for external radiotherapy by defining a biological target volume.^13^ In our survey, during preoperative staging of the disease, RO who treated ≤5 patients per month used more the combination of 18-FDG PET/CT and contrast-enhanced MRI compared with >5 patients per month. Here, we emphasized that the experience of ROs should be increased in RSJ region cancers, especially in MRI imaging. In our daily practice, we still hesitate to determine the local region with MRI only excluding 18-FDG PET/CT, even at the stages not recommended in the guidelines.

A single definition for the localization of the RSJ has not yet been reported. Eventually, an easily reproducible definition should be found by RO for the formal identification of RSJ and accurate indication of neoadjuvant CRT. Most definitions are based on morphological and descriptive criteria; on the other hand, functional definitions are based on differences in the vascularization and innervation of the colon and rectum.^14^ Different types of definitions exist in the literature before planning RT.^15^ Nevertheless, a variable distance from the anal verge is proposed worldwide. German guidelines, TNM staging, and SEER staging submit 16 cm as the upper limit of the rectum, whereas 15 cm has been proposed by the United States (ASCRS), United Kingdom, European guidelines (ESMO), and the UICC Manual. Other guidelines include a distance of 12 cm (Spanish guidelines) and 9 cm (Korea).^16^

Colonoscopy has been widely used and consists of the measurement from the anal verge to the proximal tumor edge. High specificity for the detection of rectal cancer has been described with this technique.^15^ Despite its high specificity, the distance of a tumor from the anus can vary due to loops and curves, even during the same procedure. Loffeld et al^17^ compared the accuracy of colonoscopy to radiological imaging in patient groups resected and histologically confirmed in the specimen. They concluded that if the tumor was surrounded by serosa, then the cancer was sigmoid; if the tumor was surrounded by perirectal fat without a serosal surface, it was rectal cancer. Finally, if the frontal edge of the tumor showed serosa and the dorsal plane perirectal fat, the tumor was located in the “rectosigmoid” or the pelvic part of the rectum. The methods of treatment were chosen via only colonoscopy in 66 (50.8%) responses. It was revealed that 45 (34%) participants made decisions based on both colonoscopy and MRI images in the pretreatment evaluation. One of the points that this study wanted to emphasize is that the results of these 2 examinations can be contradictory, and in this case, which examination is more preferred in treatment planning. According to the answers given by the participants in our study, it was seen that they could not define a common approach between colonoscopy reports and radiologic examinations.

At the same time, different approaches may be preferred during radiotherapy contouring, even in cases where a preoperative treatment decision is made based on colonoscopy in pretreatment imaging. The vast majority of participants (40.9%) explained that they would continue the preoperative treatment plan by drawing the target volumes up to the safety limit according to the upper limit of the tumor. In comparison, 37 (29.1%) would prefer surgery first because it is in the parts outside the pelvis, and 34 (26.8%) would determine the treatment plan according to whether most of the tumor’s volume is inside the pelvis or outside.

Diffusion-weighted MRI has been widely used in tumor diagnosis, tumor characterization, and monitoring response to treatment and has been included in standard protocols by providing practical information about tumor cellularity and cellular membrane integrity.^18^ An early increase in mean tumor apparent diffusion coefficient (ADC) and a low pretherapy mean ADC in rectal carcinoma have been shown to correlate with an excellent response to CRT.^19^ According to our survey, diffusion-weighted MRI was used for 56 (43%) respondents considering better treatment response and tumor volume delineation.

During the COVID-19 pandemic period, there was intensity and commotion in hospitals worldwide. In this period, there was mostly no difference in the RSJ treatment protocols in Turkey, according to our study. Few participants (10.9%) stated that they considered surgery primarily because of the contradiction in the definition of the tumor region, and very few (6.9%) described a tendency to hypofractionated RT. In contrast to the present study, Clifford et al^20^ reported that there was a rapid shift to hypofractionated RT regimens with short-course radiotherapy (SCRT), and also, there was an increase in the number of patients straight to surgery due to the COVID-19 pandemic.

The strengths of our analysis included survey data from experienced ROs representing many regions all over the country. To our knowledge, the present study is the first nationwide survey showing how much difference there is among ROs in the diagnosis and treatment preferences of RSJ tumors. Nevertheless, there are limitations. Our sample size of representative participants may not be adequate to conduct a standard definition and evaluate the diagnostic and therapeutic approaches to RSJ cancer.

## Conclusion

This study is the first nationwide survey to report Turkey’s clinical implementations of upper rectum and RSJ tumors. Our survey demonstrates that there is no sole approach to the RSJ among ROs in Turkey. The data, including large case numbers, must be built in randomized controlled trials to determine the most accurate treatment modality for RSJ carcinomas. Although a broadly acceptable preoperative diagnostic modality is the MRI setting, it is relatively less widely used for radiotherapy target volume definition in RT. It also requires more training for RO.

## Figures and Tables

**Table 1.** Demographic Characteristics, Preoperative Imaging Methods and Treatment Approaches

	**n**	**%**
I. Age group		
	<40	47	36.7
≥40	83	63.9
II. Participants		
	Specialist	77	58.2
	Associate professor	29	23.3
Professor	24	18.5
III. Type of center		
	University hospital	59	45.4
	Research hospital	45	34.6
	Private hospital	19	14.6
Community hospital	7	5.4
IV. Experience in rectosigmoid junction tumors		
	≤5	87	68
>5	41	32
V. Decision-making on multidisciplinary tumor boards		
	No	21	16.2
Yes	109	83.8
VI. Imaging for pre-RT staging the disease.		
	18-FDG PET/CT with MRI	108	83.1
Thorax CT with MRI	21	16.9
VII. Diffusion-weighted MRI was added to the standard MRI		
	No	74	57
Yes	56	43
VIII. Decision-making according to colonoscopy		
	No	19	14.6
Yes	111	85.4
IX. The difference between flexible and rigid colonoscopy is insignificant.		
	No	25	19.2
Yes	105	80.8
X. Decision-making according to MRI findings		
	No	11	8.5
Yes	119	91.5
XI. Decision-making according to colonoscopy reports		
	No	68	51.8
Yes	62	48.2
XII. Decision-making according to colonoscopy without minding whether the tumor is above the peritoneal reflection in radiologic imaging.		
	No	23	17.7
Yes	107	82.3
XIII. Decision-making according to pelvic MRI without minding where the tumor is located according to colonoscopy	64	49.2
	No	66	50.8
Yes		
XIV. Decision-making according to pelvic CT without minding where the tumor is located according to colonoscopy	70	53.8
	No	60	46.2
Yes		
XV. If undecided in assessing, treatment decision is made according to how far from the anal verge distance in colonoscopy without minding whether the tumor is above the peritoneal reflection in lower abdomen computed tomography (CT) and MRI.		
	No	64	49
Yes	66	51
XVI. Treatment decision on locally advanced RSJ cancer		
	Preoperative CRT-surgery	50	38.4
	Preoperative CRT-CT-surgery	42	32.3
	Surgery ± CT ± CRT	31	23.8
Short course RT + surgery + CT	7	5.5
XVII. Treatment decision on locally advanced upper rectum cancer		
	Preoperative CRT-surgery	55	42
	Preoperative CRT-CT-surgery	43	33
	Surgery ± CT ± CRT	21	16.4
Short course RT + surgery + CT	11	8.6
XVIII. RT technique		
	IMRT	14	10.7
	VMAT	92	71
3DCRT	24	18.3
XIX. During the RT contouring, different approaches were taken into consideration according to the tumor region		
	If target volumes extended out of the true pelvis, it was continued by expanding upper safety margins toward the colon according to the tumor region	52	40.9
	Being the most of tumor volume was closer to colon, the patient should have been directed to surgery	37	29.1
Decision-making the treatment according to whether most of the tumor’s volume is inside the pelvis or outside	34	26.8
XX. Any difference in treatment approaches during COVID-19 pandemic		
No	106	82.2
Yes—surgery is the first choice due to the tumor being already in a contradictory region such as the RSJ.	13	10.9
Yes—hypofractionated RT	11	6.9

18-FDG PET, 18 fluorodeoxyglucose-positron emission tomography; COVID-19, coronavirus disease 2019; CRT, chemoradiotherapy; CT, computed tomography; MRI, magnetic resonance imaging; RSJ, rectosigmoid junction; RT, radiation therapy.
